# Supplementary material for: Dynorphin‐based “release on demand” gene therapy for drug‐resistant temporal lobe epilepsy
Source: EMBO Mol Med. 2019 Sep 5;11(10):e9963. doi: 10.15252/emmm.201809963 (PMC6783645; doi:10.15252/emmm.201809963)
Supplement: Supplementary file 1 — Appendix [file EMMM-11-e9963-s001.pdf]

Table of content:

Appendix Figure S1 page 2

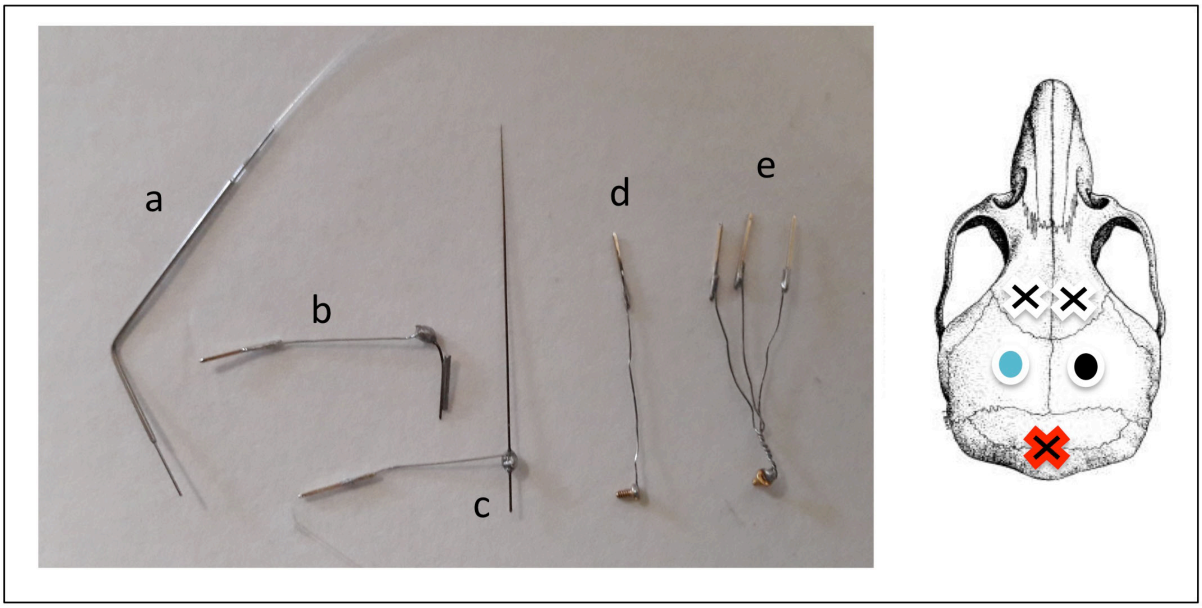

Appendix Figure S1:

Left: Infusion cannula and electrodes for EEG: The infusion canula is made from 2 steel cannulas with different diameter (a). The thinner one fits exactly into the larger one. On the upper end, a silicon tube is attached to the cannula, connecting it to the infusion pump for delivery of either KA or vector. For surgery, the depth electrode connected to the guide cannula (b) is mounted on the lower tip of the infusion cannula. The infusion cannula is mounted on one arm of the stereotaxic frame to place the guide cannula and the KA infusion into the target position. Once embedded in dental cement, this guide cannula helps to inject the vector into the same area as KA was applied. The both depth electrode are made from tungsten steel. The depth electrode for the contralateral side (c) is kept longer for mounting it to the other arm of the stereotaxic frame. After embedding in the dental cement it is clipped just above the soldering point. The surface electrodes are made from gold platted stainless steel screws (d). All electrodes are connected to gold-platted pins via stainless steel wires. The ground and reference electrodes are made from one screw connected to three pins (e).
Right: Placement of electrodes: black on white X indicate the position of the surface electrodes. The turquoise on white circle shows the site for the ipsilateral, the black on white circle the contralateral depth electrode. The black on red X indicates the position of the reference electrode
