# Supplementary material for: Dynorphin‐based “release on demand” gene therapy for drug‐resistant temporal lobe epilepsy
Source: EMBO Mol Med. 2019 Sep 5;11(10):e9963. doi: 10.15252/emmm.201809963 (PMC6783645; doi:10.15252/emmm.201809963)
Supplement: Supplementary file 2 — Expanded View Figures PDF [file EMMM-11-e9963-s002.pdf]

Expanded View Figures

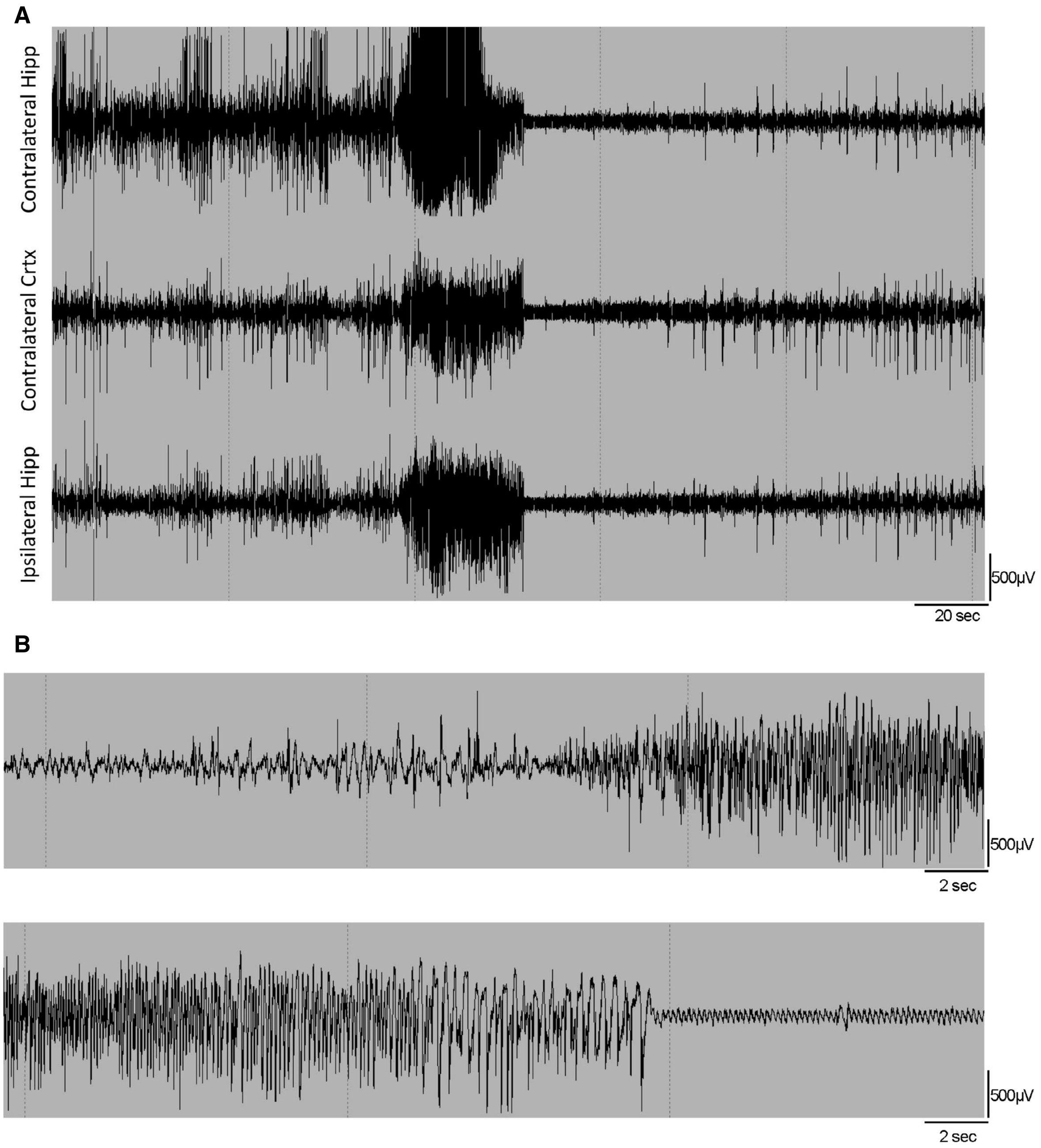

Figure EV1. EEG trace of a seizure.

A EEG trace of a generalized seizure in a KA-treated mouse; from top to bottom contralateral hippocampus, contralateral motor cortex and ipsilateral hippocampus.
B Blow-up of the EEG trace from the ipsilateral hippocampus in panel (A).

© 2019 The Authors

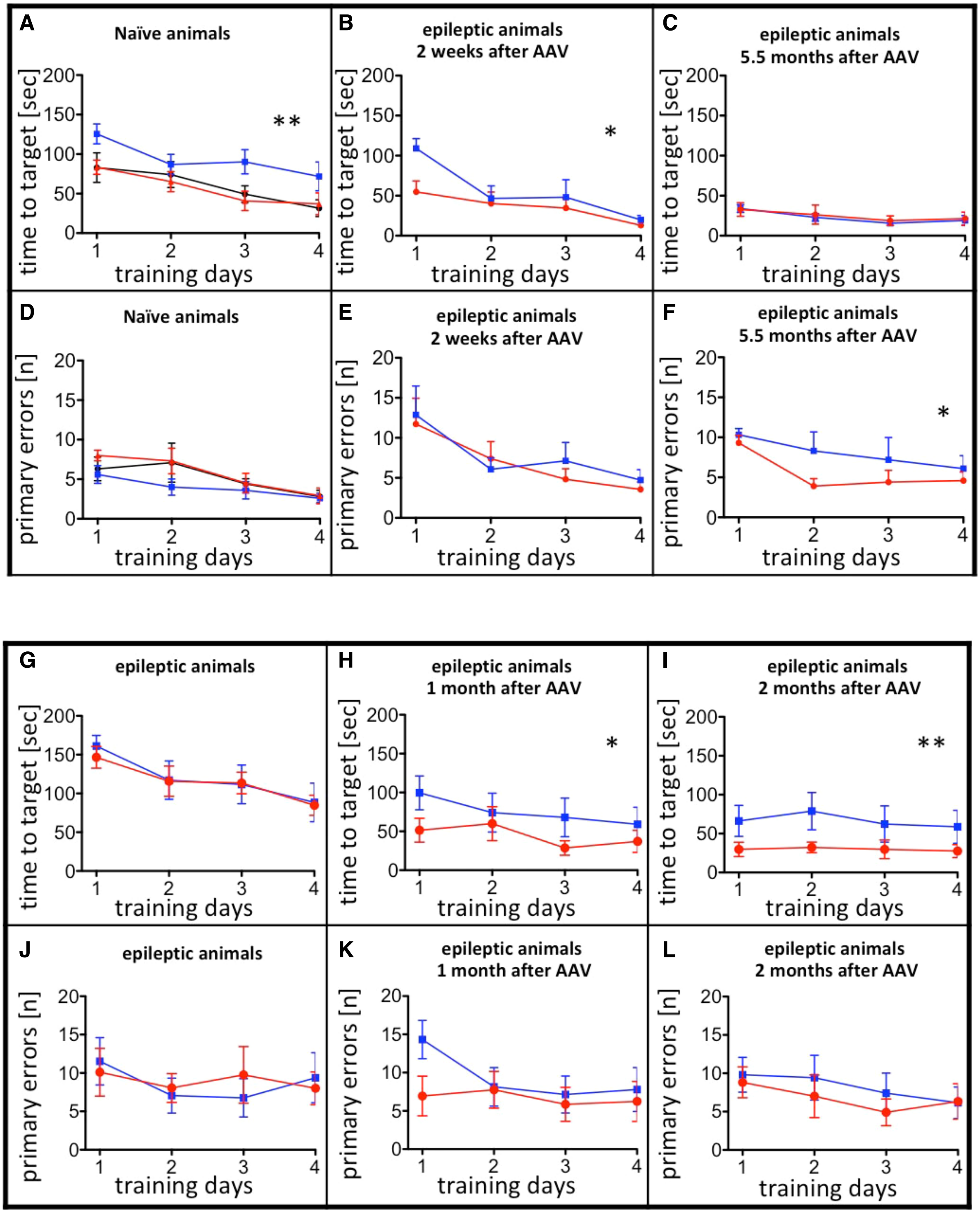

Figure EV2.

© 2019 The Authors

◀ **Figure EV2. Learning during Barnes maze testing.**

Spatial learning and memory were tested on the Barnes maze. Mice were trained on 4 consecutive days before testing memory. The acquisition of learning was monitored by assessment of the time needed to find the target hole (A–C; G–I) and the number of wrong holes visited before finding the correct one (primary errors; D–F: J–L) on each training day.

A–F Unilateral injection of AAV-pDyn (red) or AAV-ΔGFP (blue) into naïve young adult mice compared with naive controls (black) is depicted in (A) and (D). Interestingly, AAV-ΔGFP-injected mice took longer to find the hole. Noteworthy, this had no impact on memory retrieval (Fig 2A–C). Mice treated 2 weeks after KA with AAV-pDyn (red) or AAV-ΔGFP (blue) (B, C, E, F) performed differently 2 weeks after vector application (B, E) in respect to time needed to target and 5.5 months (C, F) after treatment in respect to primary errors. The overall reduction in time needed to target is most probably due to the repeated testing of animals on the Barnes maze. The target hole was repositioned in each round, but the mice were familiar with the test per se.

G–L A person not familiar with the mice assigned epileptic animals into two groups before testing on Barnes maze. No differences were observed between the two groups before vector treatment (G, J). By contrast, AAV-pDyn (red)-treated animals reached the target significantly faster than AAV-ΔGFP (blue) 1 and 2 months after treatment (H, I). Primary errors did not differ (K, L).

Data information: Animal numbers: (A) and (D) $n = 9$; (B, C, E, F) $n = 8$; for AAV-ΔGFP in (C) and (F) $n = 5$, because 3 mice had to be excluded from evaluation due to accelerating seizure activity and resulting weight loss; (G–L) $n = 7$; data represent mean ± standard error of 3 training sessions per day. $^{**}P < 0.01$; $^{*}P < 0.05$ by two-way ANOVA for repeated measurements depicting only difference between treatments. Besides the differences between treatments, significance of the factor time (i.e., the training days) was observed for data depicted in (A) ($P = 0.0002$); (B) ($P = 0.0009$); (D) ($P < 0.0001$); (E) ($P = 0.0010$); and (F) ($P = 0.0212$) suggestive of learning.

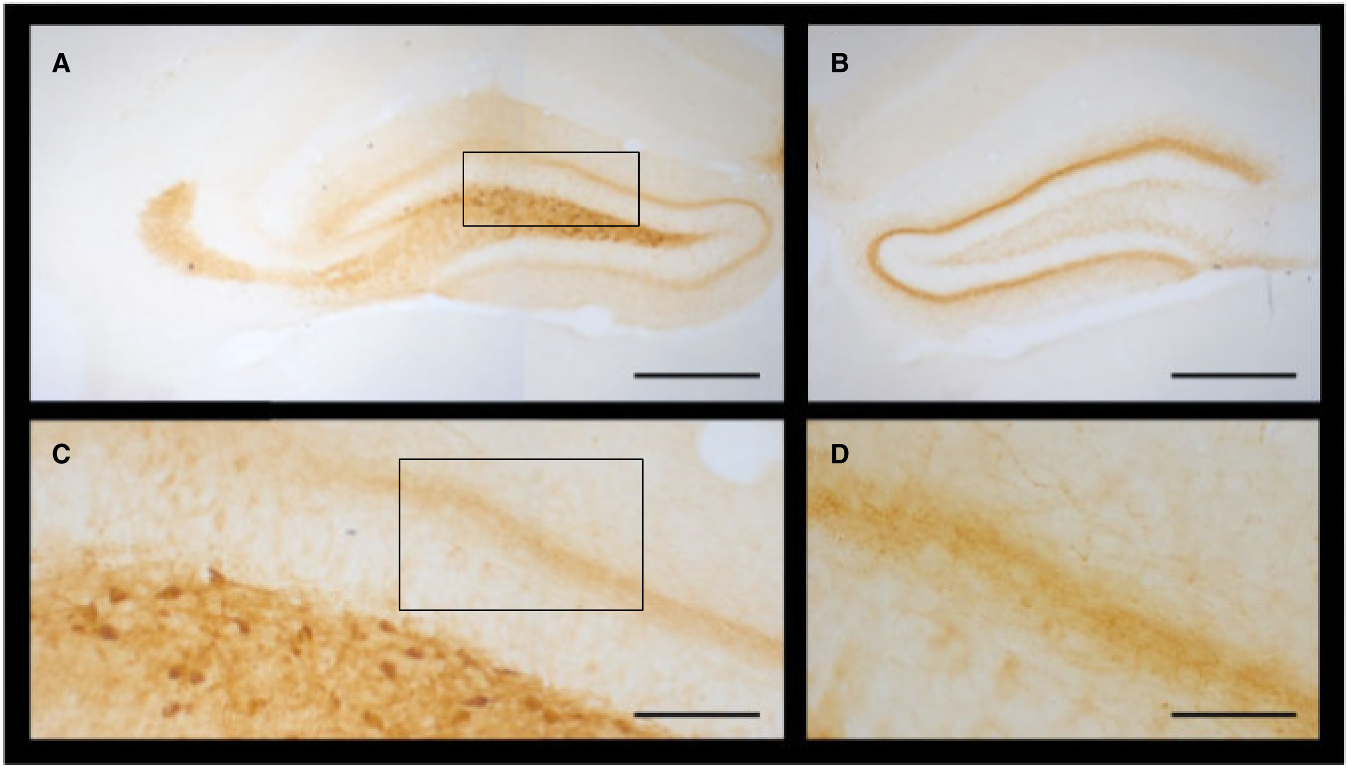

Figure EV3. Distribution of vector-derived dynorphins.

A–D Immunohistochemistry was performed on PFA-fixed 40-μm sections obtained from a pDyn knockout mouse unilaterally injected with 2×10^9 gp of AAV-pDyn 3 weeks before. The ipsilateral hippocampus (A) displays strong immunoreactivity in the terminal field of mossy fibers and the polymorph cell layer, indicative for expression in granule cells and localization in granule cell axons. Strong immunoreactivity was also observed in the inner molecular layer in both hippocampi (A, B). This region is strongly innervated by mossy cells, located in the hilus and forming strong projections also contralaterally. Several non-principal neurons were labeled in the ipsilateral polymorph cell layer (C). These cells may represent different types of GABAergic interneurons beside the glutamatergic mossy cells. Labeling in the molecular layer is dense in the supergranular band but faint in the middle and outer layers. Some well-labeled filaments (presumably fibers due to their varicose structure) are visible (D). The diffuse light labeling in the ipsilateral but not contralateral molecular layer might be due to Dyn stored in dendrites, as suggested by some studies, or represent axon terminals of GABAergic interneurons. Scale bars represent 500 μm (A) and (B); 100 μm (C); and 50 μm (D). Box in (A) delineates the section enlarged in (C). Box in (C) delineates the section enlarged in (D). Immunohistochemistry was performed using rabbit anti-human dynorphin B antibodies (Serotec AHP377; 1: 1,000) and goat anti-rabbit secondary antibodies coupled to horseradish peroxidase (Dako P 0448; 1: 400) followed by 3,3′-diaminobenzidine staining.

© 2019 The Authors

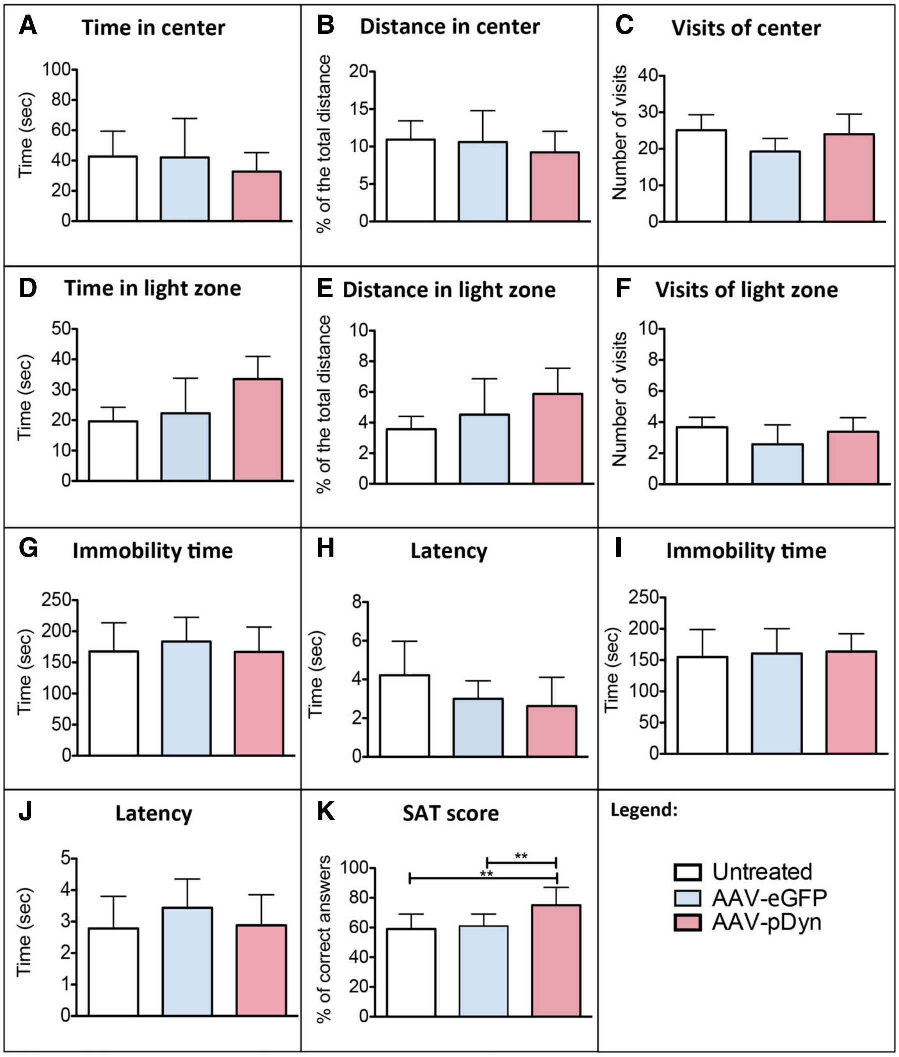

Figure EV4. Behavioral data of AAV-pDyn-injected mice.

A–K Naive mice treated with either AAV-eGFP (blue columns) or AAV-pDyn (red columns) were tested in the open-field (A–C), light–dark (D–F), forced swim (G, H) tail suspension (I, J), and spontaneous alteration (K) test. No differences were observed in anxiety (A–F) or stress-coping (G–J) behavior. AAV-pDyn-injected animals displayed a higher ratio of correct arm entries (K). $^{**}P < 0.01$, one-way ANOVA. Data represent mean ± SD of $n = 6$ per group.

© 2019 The Authors
