# Supplementary material for: Dynorphin‐based “release on demand” gene therapy for drug‐resistant temporal lobe epilepsy
Source: EMBO Mol Med. 2019 Sep 5;11(10):e9963. doi: 10.15252/emmm.201809963 (PMC6783645; doi:10.15252/emmm.201809963)
Supplement: Supplementary file 3 — Table EV1 [file EMMM-11-e9963-s003.docx]

|  | Burst Events | | | Interictal Spikes | | |
| --- | --- | --- | --- | --- | --- | --- |
|  | Amplitude | | | | | |
| Patient | Baseline | Dyn A/B | Wash/5’-GNTI | Baseline | Dyn A/B | Wash/5’-GNTI |
| 1 | 1.01 ± 0.02 mV | 0.69 ± 0.03 mV | 0.76 ± 0.03 mV | - | - | - |
| 2 | 0.44 ± 0.01 mV | 0.27 ± 0.02 mV | 0.37 ± 0.02 mV | 0.12 ± 0.0003 mV | 0.10 ± 0.0002mV | 0.08 ± 0.0004 mV |
| 3 | 0.29 ± 0.02 mV | 0.25 ± 0.02 mV | 0.4 ± 0.02 mV | 0.34 ± 0.001 mV | 0.43 ± 0.001 mV | 0.45 ± 0.001 mV |
| 4 | 0.29 ± 0.01 mV | 0.17 ± 0.01 mV | 0.37 ± 0.01 mV | 0.09 ± 0.0002 mV | 0.13 ± 0.0003 | 0.11 ± 0.0002 mV |
|  | Inter-event-intervals | | | | | |
| Patient | Baseline | Dyn A/B | Wash/5’-GNTI | Baseline | Dyn A/B | Wash/5’-GNTI |
| 1 | 1584 ± 29.6 ms | 1973 ± 76.5 ms | 3156 ± 621 ms | - | - | - |
| 2 | 3206 ± 456 ms | 8961 ± 4860 ms | 5327 ± 1516 ms | 61.9 ± 1.9 ms | 95.5 ± 2.5 ms | 445 ± 101 ms |
| 3 | 6154 ± 909 ms | 31341 ± 9303 ms | 6029 ± 738 ms | 3129 ± 9.0 ms | 216 ± 224 ms | 326 ± 345 ms |
| 4 | 3644 ± 436 ms | 7484 ± 814 ms | 3188 ± 298 ms | 1659 ± 7.3 ms | 83.4 ± 1.1 ms | 70.6 ± 1.8 ms |
|  | Number of Events | | | | | |
| Patient | Baseline | Dyn A/B | Wash/5’-GNTI | Baseline | Dyn A/B | Wash/5’-GNTI |
| 1 | 189 | 150 | 94 | - | - | - |
| 2 | 94 | 24 | 57 | 1991 | 2248 | 402 |
| 3 | 46 | 10 | 50 | 957 | 1374 | 917 |
| 4 | 82 | 46 | 86 | 1813 | 3595 | 4245 |

Table EV1:

Individual patient data obtained from slice electrophysiology on specimen removed upon epilepsy surgery. In experiments on samples from patients 1 and 2 Dyn A/B was removed for wash out. In experiments involving patients 3 and 4 the specific KOR antagonist 5’-GNTI was co-applied with Dyn A/B to demonstrate the KOR specificity of the Dyn effects.
